# Identification of alternative splice variants in *Aspergillus flavus *through comparison of multiple tandem MS search algorithms

**DOI:** 10.1186/1471-2164-12-358

**Published:** 2011-07-11

**Authors:** Kung-Yen Chang, David C Muddiman

**Affiliations:** 1 Bioinformatics Research Center, North Carolina State University, Raleigh, NC 27695, USA; 2 W.M. Keck FT-ICR-MS Laboratory, Department of Chemistry, North Carolina State University, Raleigh, NC 27695, USA

## Abstract

**Background:**

Database searching is the most frequently used approach for automated peptide assignment and protein inference of tandem mass spectra. The results, however, depend on the sequences in target databases and on search algorithms. Recently by using an alternative splicing database, we identified more proteins than with the annotated proteins in *Aspergillus flavus*. In this study, we aimed at finding a greater number of eligible splice variants based on newly available transcript sequences and the latest genome annotation. The improved database was then used to compare four search algorithms: Mascot, OMSSA, X! Tandem, and InsPecT.

**Results:**

The updated alternative splicing database predicted 15833 putative protein variants, 61% more than the previous results. There was transcript evidence for 50% of the updated genes compared to the previous 35% coverage. Database searches were conducted using the same set of spectral data, search parameters, and protein database but with different algorithms. The false discovery rates of the peptide-spectrum matches were estimated < 2%. The numbers of the total identified proteins varied from 765 to 867 between algorithms. Whereas 42% (1651/3891) of peptide assignments were unanimous, the comparison showed that 51% (568/1114) of the RefSeq proteins and 15% (11/72) of the putative splice variants were inferred by all algorithms. 12 plausible isoforms were discovered by focusing on the consensus peptides which were detected by at least three different algorithms. The analysis found different conserved domains in two putative isoforms of UDP-galactose 4-epimerase.

**Conclusions:**

We were able to detect dozens of new peptides using the improved alternative splicing database with the recently updated annotation of the *A. flavus *genome. Unlike the identifications of the peptides and the RefSeq proteins, large variations existed between the putative splice variants identified by different algorithms. 12 candidates of putative isoforms were reported based on the consensus peptide-spectrum matches. This suggests that applications of multiple search engines effectively reduced the possible false positive results and validated the protein identifications from tandem mass spectra using an alternative splicing database.

## Background

Tandem mass spectrometry (MS/MS) has been one of the most effective high-throughput approaches for protein identification and quantification. In a typical "bottom-up" approach, also known as the shotgun proteomics strategy, the enzyme-digested protein mixture is analyzed using single- or multi-dimensional chromatography coupled with tandem mass spectrometry [1,2]. A variety of computational approaches have been developed to assign peptide sequences to the acquired MS/MS data. Database searching algorithms are the most frequently used methods for large-scale proteomics studies [3]. The most popular commercial MS/MS search engines are SEQUEST [4] (Thermo Fisher Scientific Inc.) and Mascot [5] (Matrix Science Ltd.). Open source tools are also available, such as OMSSA [6], X! Tandem [7], and Andromeda [8]. Although each implementation is different, the general approach of MS/MS search algorithms is similar [9]. Given a protein sequence database, the search algorithm first generates all *in silico*-digested peptides upon the specified parameters, such as digestive enzymes, missed cleavages, and modifications. For each MS/MS spectrum, the search engine only evaluates the candidate peptide sequences within a user-defined precursor mass tolerance window. A scoring function is used to calculate a score which represents how well the theoretical spectrum of each candidate peptide matches the observed spectrum. The top scoring peptide hit is reported and then the peptide sequence is assigned to the experimental MS/MS spectrum. Protein identifications are inferred by grouping the peptide-spectrum matches [10].

Another approach for identifying peptides from fragment ion spectra combines partial *de novo *sequencing and database searching. Short peptide sequence tags are inferred from MS/MS spectra using *de novo *algorithms. The list of candidate peptides in the database search can be reduced to only those containing the tag [11]. The algorithms will then try to extend the sequence tag by finding masses of the flanking residues in the database peptide which match masses of the prefix and suffix regions of the tag [12]. Although the hybrid approach is still reliant on protein sequence databases, it is an alternative strategy while analyzing peptides with novel modifications or sequence variations [13].

Alternative pre-mRNA splicing (AS) enables eukaryotes to generate distinct mRNAs and therefore multiple protein variants from a single gene. The common approach to developing an alternative splicing database is based on automated large-scale mapping of transcripts and genomic sequences. The massively parallel picolitre-scale sequencing system developed by the 454 Life Sciences Corporation was capable of sequencing 25 million bases in a four-hour run [14]. The 454 sequence reads are short, averaging 80-120 bases per read. The massively parallel sequencing-by-synthesis technology has been used to generate EST data of a human prostate cancer cell line, and 25 novel alternative exon splicing events were identified [15].

**Figure 1:**
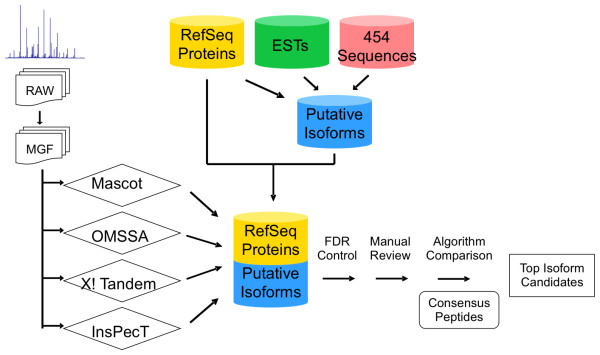
**Schematic of study design**.

Recently, we expanded the target database to include putative alternatively spliced isoforms with the aim that the MS/MS spectra can be better interpreted [16]. The results showed that our approach was able to identify more proteins from the experimental spectra and to provide evidence for improving the genome annotation. Subsequently, the *Aspergillus flavus *NRRL3357 whole genome shotgun project had a major update in 2009. Among 41 peptides discovered in our previous study, 6 of them were included in the second version of genome annotation. Meanwhile, 454 sequencing data of *A. flavus *became available locally. The first goal of this study was to rebuild the alternative splicing database using the latest genome annotation and newly acquired 454 sequencing data as transcript evidence. The second part of the study aimed at comparing four MS/MS search algorithms for isoform identifications using the resulting alternative splicing database. We tested three probability-based algorithms, Mascot [5], OMSSA [6], and X! Tandem [7], and one sequence tag-based algorithm, InsPecT [12]. The design of the study is illustrated in Figure 1.

## Results

### Rebuilding *A. flavus *alternative splicing database

**Figure 2:**
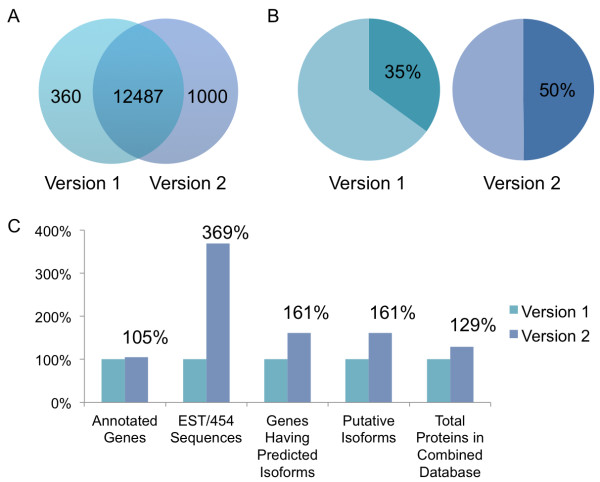
**Comparison of different versions of *A. flavus *genome**. **(A) **The latest genome contained 13487 genes. 360 prior genes were dropped and 1000 novel ones were added. **(B) **Half of the latest genes found the matched ESTs and/or 454 sequence reads. **(C) **The improved alternative splicing database showed 61% more genes having predicted splice variants and an increase of 29% in database size.

Genome annotation is the result of continuous efforts. An updated version of *A. flavus *genome annotation was released in 2009. Compared to the prior genome project, the second version dropped 360 previously documented genes and added 1000 novel ones (Figure 2A). A newly acquired collection of 454 sequence reads and ESTs provided the transcription information of half of the genes for predicting splice variants (Figure 2B). An updated alternative splicing database was then built using the second version of the genome and all available transcripts. The RefSeq database (release 40) contained 13487 *A. flavus *genes and corresponding proteins, with no splice isoform. The updated alternative splicing database predicted another 15833 putative protein variants (Figure 2C). It was estimated that 15.4% (2077/13487) of the total genes encoded more than one protein, 7.62 (15833/2077) putative isoforms per gene on average. The predicted variant sequences were appended to the collection of the RefSeq proteins to form a combined database for the following database searches.

### Comparison of MS/MS search algorithms on identifying putative isoforms

In order to compare the performance of identifying putative splice variants, the same set of MS/MS spectra were searched against the resulting combined database by Mascot, OMSSA, X! Tandem, and InsPecT. Although each algorithm already reported internal statistical measures like *p*-value or *E*-value, the cut-off thresholds were selected to ensure the search results had an estimated false discovery rate (FDR) < 2% for peptide identification (see Additional file 1). While several isoforms were encoded from the same gene, sometimes the different protein products could not be distinguished by the identified peptides. In such a scenario, it was observed that Mascot would pick the protein with the longest sequence from all possible candidates. InsPecT would also report one protein from the list of candidate sequences, but not necessarily the longest one. In contrast, OMSSA and X! Tandem would report all matched proteins and let users interpret the findings. In order to present the results concisely, we accepted the longest protein sequence to represent the group of all possible matches. If a group of peptides could be mapped to either the RefSeq protein or the splice variant of the same gene, we conservatively assigned the identification to the RefSeq protein since no clear conclusion was possible. The number of identified peptides, RefSeq proteins, and splice variants by algorithms are listed in Table 1.

**Table 1:** Number of identified peptides and proteins by algorithms with a FDR < 2%

Algorithm	Threshold	Number of Identified Peptides	MS/MS FDR (%)	Number of Identified RefSeq Proteins	Number of Identified Splice Variants
Mascot	*E*-value < 0.001	2731	1.88	747	21
OMSSA	*E*-value < 0.09	2407	1.89	745	20
X! Tandem	*E*-value < 0.04	2682	1.76	831	36
InsPecT	*p*-value < 0.02	2563	1.91	738	33

**Figure 3:**
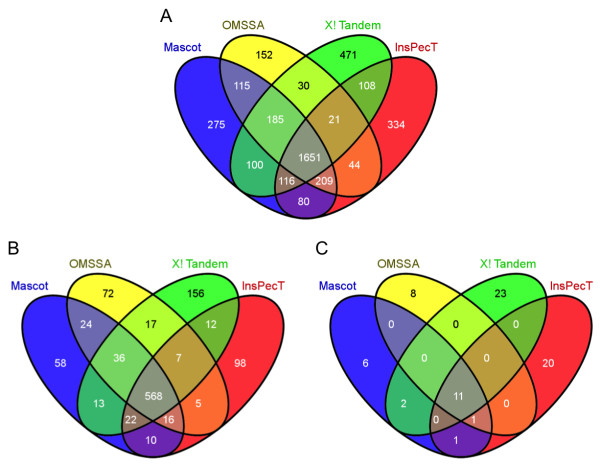
**Overlap of peptide and protein identifications using different search algorithms**. The 4-way Venn diagrams generated by the VENNY program [33] illustrate the intersections of **(A) **all peptides, **(B) **RefSeq proteins, and **(C) **putative splice variants identified by Mascot, OMSSA, X! Tandem, and InsPecT. In addition to 42% (1651/3891) of identified peptides overlapping, all four algorithms agreed on 51% (568/1114) of RefSeq protein identifications but only 15% (11/72) of the putative splice variants. All search results had an estimated FDR < 2% for peptide identification.

To study the consistency between different algorithms on search results, the identified hits were categorized by the algorithms having the same finding (Table 2). The overlaps were illustrated in four-way Venn diagrams as well (Figure 3). For the peptide-spectrum matches, 42% (1651/3891) of peptide assignments were concurred by all four algorithms. Since we introduced predicted isoform sequences into the database, the protein identification was divided into two subgroups: RefSeq proteins and putative splice variants. 51% (568/1114) of the identified RefSeq proteins were consistent across all algorithms. In contrast, only 15% (11/72) of the putative splice variants were identified unanimously.

**Table 2:** Overlap of identified peptides and proteins between algorithms with a FDR < 2%

Algorithm	Peptides		RefSeq Proteins		Putative Isoforms	
			
	Count	%	Count	%	Count	%
Mascot only	275	7.1	58	5.2	6	8.3
OMSSA only	152	3.9	72	6.5	8	11.1
X! Tandem only	471	12.1	156	14.0	23	31.9
InsPecT only	334	8.6	98	8.8	20	27.8
Mascot, OMSSA	115	3.0	24	2.2	0	0.0
Mascot, X! Tandem	100	2.6	13	1.2	2	2.8
Mascot, InsPecT	80	2.1	10	0.9	1	1.4
OMSSA, X! Tandem	30	0.8	17	1.5	0	0.0
OMSSA, InsPecT	44	1.1	5	0.4	0	0.0
X! Tandem, InsPecT	108	2.8	12	1.1	0	0.0
Mascot, OMSSA, X! Tandem	185	4.8	36	3.2	0	0.0
Mascot, OMSSA, InsPecT	209	5.4	16	1.4	1	1.4
Mascot, X! Tandem, InsPecT	116	3.0	22	2.0	0	0.0
OMSSA, X! Tandem, InsPecT	21	0.5	7	0.6	0	0.0
Mascot, OMSSA, X! Tandem, InsPecT	1651	42.4	568	51.0	11	15.3
Grand Total	3891	100.0	1114	100.0	72	100.0

To investigate whether different algorithms assigned the same spectrum to different peptide sequences, the peptide-spectrum matches were examined within and between algorithms (Table 3). It was observed for all algorithms that 1% or fewer spectra were assigned to different peptides by the same tool. The inconsistency expanded but never exceeded 2% while comparing the assignment of the same spectrum between different algorithms. It also appeared that InsPecT assigned more spectra differently in comparison with other three probability-based algorithms. The multiple peptides assigned from the same spectra between algorithms might account for a part of the identification variations.

**Table 3:** Number of MS/MS spectra assigned to different peptide sequences by algorithms

Algorithm	Number of assigned spectra	Assigned to different peptides by Mascot	Assigned to different peptides by OMSSA	Assigned to different peptides by X! Tandem	Assigned to different peptides by InsPecT
Mascot	9410	87 (0.92%)	134 (1.42%)	77 (0.82%)	132 (1.40%)
OMSSA	8531	128 (1.50%)	64 (0.75%)	77 (0.90%)	137 (1.61%)
X! Tandem	8820	76 (0.86%)	76 (0.86%)	26 (0.29%)	116 (1.32%)
InsPecT	8832	132 (1.49%)	134 (1.52%)	116 (1.31%)	93 (1.05%)

It was not surprising to see that the number of peptide-spectrum matches and protein hits dropped while reducing the false discovery rate. However, most of the removed hits belonged to the identifications reported by only one algorithm (see Additional file 2). The consensus hits of multiple algorithms seemed more likely to be the correct identification. In the comparison of the overlaps between search results, the identified splice variants between different algorithms showed greater variations than the RefSeq proteins. It is noted that the prediction of all possible splice variants from ESTs tends to be over-estimated. To reduce the false positive results, we compiled a list of top splice isoform candidates by taking advantage of the consensus peptides. By focusing on those variant-specific peptides identified by at least three different algorithms, 12 putative isoforms were reported (Table 4). 11 splice variants were inferred by all four algorithms. The scores, *p*-values, and *E*-values of the assignments looked satisfying. None of these specific peptide sequences appeared in any RefSeq proteins. In addition, no two consensus peptides came from the same spectra. As an example, one putative isoform discovered through the strategy was further analyzed below.

**Table 4:** List of consensus peptides specific to putative isoforms with a FDR < 2%

Gene ID	Gene Description	Peptide Specific to Putative Isoform	Mascot Prot Score	Mascot Pep E-value	OMSSA E-value	OMSSA p-value	X!Tandem Prot Expect	X!Tandem Pep Expect	InsPecT MQ Score	InsPecT p-value
7910490	prefoldin subunit 6	AEILQYQSQMQQQAAAASASA	69	3.1E-06	4.2E-04	1.6E-06	n.a.	n.a.	0.921	4.8E-03
7912171	peroxiredoxin	VENNDILFLSDPDAK	145	1.1E-09	2.8E-09	1.1E-11	-7.7	8.5E-04	n.a.	n.a.
		VSGAEAVLAHL	145	6.6E-07	6.2E-06	6.1E-08	-7.7	1.6E-02	2.728	1.0E-05
7914158	hypothetical protein	ENALEAGQVVAVLAEGK	187	1.1E-10	4.7E-12	1.9E-14	n.a.	n.a.	3.375	1.0E-05
		LPEKENALEAGQVVAVLAEGK	187	4.3E-05	1.4E-05	1.3E-07	-3	9.3E-04	n.a.	n.a.
7914461	UTP-glucose-1-phosphate uridylyltransferase Ugp1	APATETSNAGSFGK	296	2.5E-09	2.0E-04	1.0E-06	-15.6	5.0E-05	2.791	1.0E-05
7914540	conserved hypothetical protein	EFEDAAFALQPGQVSGIVDTASGVHLIER	109	3.2E-06	2.1E-07	6.4E-10	-7.2	4.2E-03	n.a.	n.a.
		SKEEAIEILR	109	1.4E-04	4.3E-03	1.7E-05	-7.2	1.0E-02	1.705	1.0E-05
7916030	cyclophilin	SGELESEDKGSHEEL	216	4.0E-05	2.4E-03	2.8E-05	-1.7	2.0E-02	2.184	1.0E-05
7918378	14-3-3 family protein ArtA	EEAPAAEGEKPAAE	380	1.0E-07	1.5E-04	9.8E-07	-27.3	4.5E-04	1.901	1.0E-05
		KEEAPAAEGEKPAAE	380	2.8E-11	4.2E-08	1.7E-10	-27.3	6.8E-07	2.991	1.0E-05
7919242	conserved hypothetical protein	VADVGTGTAIWLTDLAK	130	1.3E-09	1.6E-10	1.4E-12	-9.9	1.3E-05	3.067	1.0E-05
7919622	phosphofructokinase	NDQTSTIYSTTEIANIIK	61	4.1E-06	2.0E-06	1.2E-08	-3.4	3.9E-04	n.a.	n.a.
7919639	UDP-glucose 4-epimerase	FAVETAITDVINAQR	710	1.8E-12	2.7E-10	1.5E-12	-25.4	2.4E-06	2.187	1.0E-05
7919713	14-3-3 protein sigma, gamma, zeta, beta/alpha	DNLTLWTSSDGQEPEGAASK	129	6.8E-13	5.1E-12	2.8E-14	-8.3	5.3E-09	3.447	1.0E-05
7920463	ubiquinol-cytochrome C reductase complex core protein 2	FLSNDLPYFAELLAEVASQSK	131	3.6E-07	2.9E-09	1.3E-11	-13.4	1.4E-03	2.754	1.0E-05

### Conserved domain analysis of putative isoforms of UDP-galactose 4-epimerase

**Figure 4:**
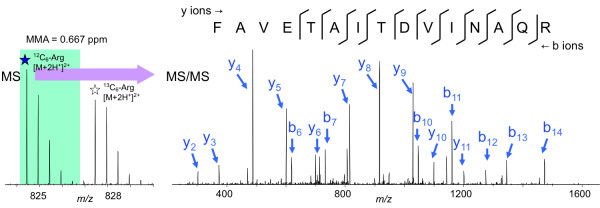
**Identification of consensus peptide FAVETAITDVINAQR**. The MS/MS spectrum of peptide FAVETAITDVINAQR which was specific to the splice variant of *A. flavus *UDP-glucose 4-epimerase [Entrez Gene: 7919639] resulted from a 2^+ ^precursor ion at *m/z *824.44 with a measured mass accuracy of 0.667 ppm. The MS spectrum showing a SILAC pair of ^12^C_6_-Arg (*m/z *= 824.44) and ^13^C_6_-Arg (*m/z *= 827.45) peptides with a 3 Da mass difference supported the identification, since an arginine appeared on the C terminus of the peptide sequence.

UDP-glucose 4-epimerase (UGE) [KEGG: EC 5.1.3.2] plays a pivotal role in normal galactose metabolism, converting UDP-galactose back to UDP-glucose in the final step of the Leloir pathway [17]. NAD^+ ^is required to be a cofactor in the catalytic mechanism. Five UGE isoforms encoded in the *Arabidopsis thaliana *genome differed in enzymatic properties, transcript regulation, and subcellular localization [18]. The MS/MS spectrum which was used to assign the consensus peptide FAVETAITDVINAQR in the putative UGE isoform was examined (Figure 4). The abundant matched b- and y- ions, accurate precursor ion mass, and expected mass difference from the SILAC pair observed in the spectrum correlated well with the low expectation value or *p*-value reported by algorithms.

**Figure 5:**
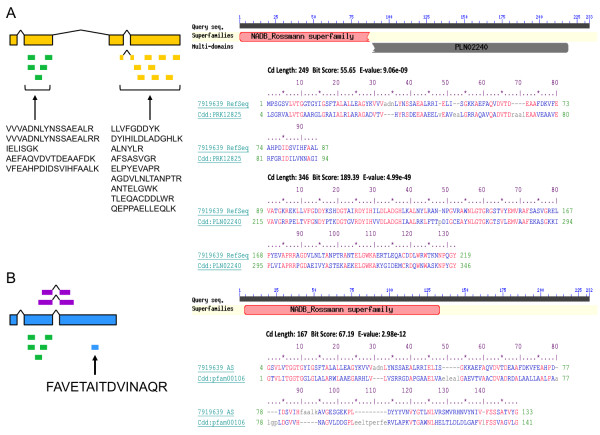
**Conserved domain analysis of *A. flavus *UDP-glucose 4-epimerase isoforms**. **(A) **The RefSeq protein of the UDP-galactose 4 epimerase consisted of four exons. The first two were constitutive exons. All Mascot, OMSSA, X! Tandem, and InsPecT confirmed the existence of the RefSeq protein based on different numbers of shared and RefSeq-specific peptides. The 5 common and 9 RefSeq-specific peptides detected by Mascot are illustrated. Two functional domains, 3-ketoacyl-(acyl-carrier-protein) reductase [CDD: PRK12825] and UDP-glucose 4-epimerase [CDD: PLN02240], were recognized through searching the sequence against the Conserved Domain Database (version 2.23) and an *E*-value threshold of 0.01. **(B) **Peptide FAVETAITDVINAQR was used to conclude the alternative exon in the putative isoform. Short chain dehydrogenase [CDD: pfam00106] domain was found in the sequence of the alternatively spliced variant via searching the CDD database. Mapped 454 sequence reads are labeled in purple. RefSeq protein-specific, splice variant-specific, and commonly shared peptides are labeled in yellow, blue and green, respectively.

According to the annotation of RefSeq release 40, *A. flavus *UDP-glucose 4-epimerase [Entrez Gene: 7919639] contained four coding exons (Figure 5A). The corresponding splice variant generated from our prediction had three exons instead: the first two were constitutive and the third was alternative (Figure 5B). Since different sets of peptide-spectrum matches were used to conclude the protein identification between search algorithms, the peptides shown in Figure 5 are based on Mascot's result. The alternative exon in the protein variant was supported by the distinctive peptide FAVETAITDVINAQR which was located in an intron of the corresponding RefSeq protein. The encoding variant sequence ended approximately in the middle of the third coding exon of the RefSeq counterpart. A group of 9 peptides which were mapped to the remaining coding sequence supported the identification of the RefSeq protein.

While multiple protein products are encoded from the same gene, different isoforms are usually destined for performing various biological functions. Thus, we were interested in learning whether two identified UGE isoforms had different functional motifs among their sequences. The Conserved Domain Database (CDD), part of NCBI's Entrez database system, is a protein annotation resource that consists of a collection of well-annotated multiple sequence alignment models as position-specific score matrices (PSSMs) [19]. Two motifs were found by searching the RefSeq sequence against CDD (version 2.23, containing 37407 PSSMs) (Figure 5A). One was a member of the Rossmann-fold NAD(P)(+)-binding proteins superfamily, 3-ketoacyl-(acyl-carrier-protein) reductase [CDD: PRK12825], and the other was UDP-glucose 4-epimerase [CDD: PLN02240]. A different member of the Rossmann-fold NAD(P)(+)-binding proteins superfamily, short chain dehydrogenase [CDD: pfam00106], was found in the sequence of the alternatively spliced variant (Figure 5B). UDP-galactose 4-epimerase is known as a member of the short chain dehydrogenase/reductase superfamily. These enzymes contain a conserved Tyr-X-X-X-Lys motif necessary for catalytic activity. The characteristic YXXXK motif of human epimerase was located at Tyr-157-Gly-Lys-Ser-Lys-161 [20]. The YXXXK signature sequence, Tyr-156-Gly-Asn-Thr-Lys-160 (YGNTK), was also found in the predicted variant sequence of *A. flavus *UGE. The different sets of motifs found in two UGE proteins suggested the putative isoforms may carry out different functions *in vivo*.

## Discussion

A new *A. flavus *alternative splicing database was rebuilt referencing the latest genome annotation. By incorporating new qualified 454 sequence reads, more splice variants were predicted from more genes compared to the previous database. Though several previously discovered peptides had been included in the updated proteome, newly predicted variants were identified from the improved database using the same set of spectra. According to the Mascot results, 29 additional proteins from 26 genes were found in the previous study [16] while the 21 putative isoforms encoded by 21 genes were reported in this study. The results suggested that the increase of transcript sequences was able to predict eligible splice variants though the genome had been updated recently.

Different groups have conducted comparative evaluations of MS/MS search algorithms [9,21]. The variation in scoring functions and statistical significance techniques in database-searching algorithms give different identification results. The overlaps of the search results from multiple algorithms can shift significantly as search parameters are modified [22]. However, those studies were performed using general protein databases without emphasizing alternatively spliced isoforms. In this study, Mascot, OMSSA, X! Tandem, and InsPecT were compared using an alternative splicing database. In spite of the agreement on 42% of peptide and 51% of RefSeq protein identifications, our results showed that 15% of the putative splice isoforms were detected by all algorithms (Table 2). The fact that less than 2% of spectra were assigned to multiple peptide sequences did not explain all the variation in isoform identifications (Table 3).

**Figure 6:**
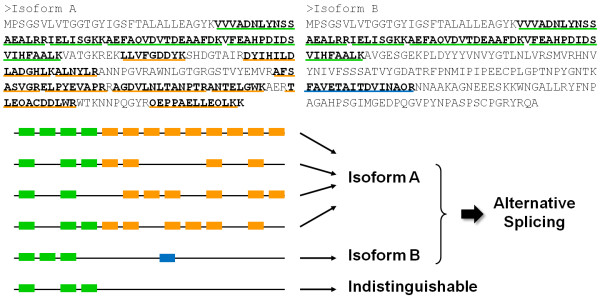
**Detection of isoform-specific peptides plays a critical role in identifying alternatively spliced isoforms**. The finding of two putative isoforms of *A. flavus *UDP-galactose 4 epimerase is illustrated as an example. Isoform A (RefSeq protein) and Isoform B (putative splice isoform) were identified by shared (green) and unique (yellow/blue) peptides. The identifications of both Isoform A and B are needed to declare the occurrence of an alternative splicing event. The detection of the splice isoform-specific (blue) peptide is decisive for the identification of Isoform B. Since only one isoform-specific peptide was found in this example, a false positive or missed identification of the peptide could alter the result of the isoform detection.

To be cautious, we chose the RefSeq protein to represent a protein group when there was no decisive peptide belonging to the putative isoforms. This allowed different algorithms to assign various peptide groups to the same RefSeq protein, thus might indirectly increase the RefSeq protein identifications. On the other hand, the inference of isoform detection mainly relied on identifying the unique peptides which exclusively belonged to variant sequences (Figure 6). As a result, the difference in peptide identifications might lead to a greater variation in isoform identifications. The variation between the splice variants identified by different algorithms implied that many unique peptides concluded by one algorithm were not necessarily recognized by another. Especially when the existence of putative isoforms was suggested by one or two isoform-specific peptides, an incorrect identification or missed detection of the specific peptides can change the conclusion immensely. Combination of multiple MS/MS search methods was used to distinguish the correct peptide identifications from the incorrect [23] and improve peptide identification rates [22]. We took advantage of consensus peptides assigned by at least three algorithms to generate 12 top candidate isoforms from the search results having estimated FDRs < 2% (Table 4). A recent study showed that the error rate of peptide hits was effectively reduced to 0.5% when a minimum of three engines were used [24]. The multiple search engine approach for peptide assignment not only takes advantage of differences in scoring functions to expand the target space for searching, but also bolsters the confidence of the peptide identifications [24].

## Conclusions

The prediction of the alternatively spliced variants based on EST sequences by a computational pipeline inclines to be over-estimated and may contain errors. The introduction of putative isoforms into the protein database can further lower the *p*-value of peptide identifications because of the increasing size of the database. Consensus decision making exploits the goodness of multiple search algorithms to validate the assignment results of spectral data at a relatively low cost. The approach is particularly valuable while making inferences in isoform identifications from an alternative splicing database.

## Methods

### RefSeq Proteins

The *A. flavus *NRRL3357 whole genome shotgun project [Refseq: NZ_AAIH00000000] released an updated version on Aug 12, 2009. The second version of the project contains 13487 genes and coding proteins, and no splice isoforms were included in the genome annotation. The nucleotide records and protein sequences of *A. flavus *NRRL3357 were downloaded from RefSeq release 40 (March 7, 2010) using Taxonomy ID equal to 332952. Other supplementary information including coding exons was collected from Entrez Genome and Entrez Gene databases.

### Alternative Splicing Database

The alternative splicing database of *A. flavus *in this study was constructed using the most recent official version of the genome described above. Serving as transcription evidence, 21130 EST sequences and 559014 454 sequences were used to predict putative slicing variants. 20371 ESTs were downloaded from the EST database of NCBI by specifying the species "*Aspergillus flavus*". All 454 sequences and an additional 759 ESTs were provided by the Center for Integrated Fungal Research at North Carolina State University.

The EST and 454 sequences were first mapped to the annotated gene sequences using BLAST [25] (version 2.2.22). To ensure the quality of the predicted splicing variants sequences, only those EST/454 transcripts which satisfied the threshold (*E*-value < 0.001) were aligned against the corresponding genes by sim4 [26]. The alignments were allowed to search 3000 bases upstream and downstream to capture any potential missing exons. The distance of 3 kb was decided as two times the length of the largest intron found in the current genome annotation. For each gene, all splice sites of exons reported by sim4 alignments were integrated into a data structure called a splicing graph [27]. In the resulting directed graph, edges represented putative exons, vertices stood for splice sites, and paths denoted transcripts. If more than one exon (edge) pointed to the same 5' splice site (vertex) or the same 3' splice site (vertex) followed by multiple possible exons (edges), alternative splicing events were indicated. The putative splicing variants from the same gene were generated by visiting all possible paths. The corresponding protein sequences were translated from the predicted transcripts with a minimum length requirement of eighteen amino acids. Finally, any predicted protein whose sequence was either a subsequence or an identical duplicate of one entry in the RefSeq database was removed before conducting the database searches.

### Experimental Spectra

The MS/MS spectra used in this study were generated in a previous experiment [28]. In brief, ^12^C_6_-Arg and ^13^C_6_-Arg labeled cultures of *A. flavus *were grown for 24 h at 28°C or 37°C. Extracted protein samples were separated on 12.5% SDS-PAGE gel. Forty bands from each lane were excised then they were reduced, alkylated, and digested by trypsin for 18 h at 37°C. Each of the 40 in-gel digested samples was analyzed by nanoflow LC-MS/MS on a LTQ-FT (ThermoFisher Scientific). The bottom-up SILAC *A. flavus *data associated with this manuscript may be downloaded from the Proteome Commons Database [29] Tranche network using the following hash: O9h2YUGGpAOG+ex5+rYTySoRxqvyPayGlWPspibKkA13BXCVcpVMp3oCmH4HwZOofp5azAQcx4coCH6I82DCx5vQjwwAAAAAAAAn5g==.

### Database Search

Four different MS/MS search algorithms were chosen for comparison, including Mascot Server (version 2.2.04) from Matrix Science Ltd., OMSSA (version 2.1.7) from NCBI, X! Tandem TORNADO (2010.01.01.4) from the Global Proteome Machine Organization, and InsPecT (version 20100804) from the Center for Computational Mass Spectrometry at the University of California, San Diego. The original spectra were stored as Thermo XCalibur RAW files. To ensure that all four search algorithms started with the same set of peak lists, the experimental spectra in RAW file format were first converted to the files in Mascot Generic Format (MGF) by Mascot Distiller (Matrix Science Ltd.) using the same processing options. A total of 311105 spectra from 77 MGF files were used in this study. The database searches were performed with the same parameters for all four search algorithms. The settings specified trypsin as the protease, a maximum of two missed cleavage sites, precursor charge up to 3^+^, 5 ppm precursor ion tolerance (0.01 Da for OMSSA), and 1 Dalton product ion tolerance. The searches also accounted for carbamidomethyl modification on Cysteine (C) as a fixed parameter, and variable modifications included oxidation on Methionine (M) and deamidation on Asparagine (N) or Glutamine (Q). This study focused on detecting splice isoforms instead of exploring the protein profiles at different temperatures. Although the input spectra were derived from a previous SILAC experiment, the data were only searched for light peptides without the ^13^C_6_-Arg label. It is noted that the setting of the refinement node for X! Tandem is ON as default.

### False Discovery Rate

The FDR for each search result was estimated through searching the decoy (reverse) database and then counting the number of peptide-spectrum matches identified from the target database (N_t_) and decoy database (N_d_). The target-decoy database search can be conducted in two ways: a single search against a concatenated target/decoy database; or two independent searches against the target and decoy databases, respectively. The separate search provided a conservative estimate [30]. FDRs of the peptides identified by Mascot, OMSSA, and X! Tandem were estimated using the separate search strategy and calculated as N_d_/N_t _[31]. However, the separate search approach was not feasible for the InsPecT results. The InsPecT tutorial describes that most results are not statistically significant and post-processing is essential. It is necessary to run the PValue.py script, included in the InsPecT distribution, to weed out insignificant results. The script uses a concatenated target/decoy database to calibrate the *p*-value by fitting the score distribution with a mixture model. Hence, FDR of the peptides identified by InsPecT was estimated using the concatenated database strategy instead, computed as 2 * N_d_/(N_t _+ N_d_) [32].

## Competing interests

The authors declare that they have no competing interests.

## Authors' contributions

KYC carried out the construction of alternative splicing database, performed database searches and the statistical analysis, and drafted the manuscript. DCM conceived of the study, participated in its design and coordination, and helped draft the manuscript. All authors read and approved the final manuscript.

## Supplementary Material

Additional file 1 **Calculation of MS/MS FDRs**. The steps for deriving the false discovery rates of peptide identifications by different search algorithms are presented here in detail. Click here for file

Additional file 2 **Comparison of overlapping identifications at different FDRs**. Consensus decision of multiple search algorithms reached the similar overlaps regardless of the search results having the controlled or uncontrolled MS/MS FDRs. Click here for file
