# Supplementary material for: Identification of alternative splice variants in Aspergillus flavus through comparison of multiple tandem MS search algorithms
Source: BMC Genomics. 2011 Jul 11;12:358. doi: 10.1186/1471-2164-12-358 (PMC3146456; doi:10.1186/1471-2164-12-358)
Supplement: Additional file 1 — Calculation of MS/MS FDRs. The steps for deriving the false discovery rates of peptide identifications by different search algorithms are presented here in detail. [file 1471-2164-12-358-S1.PDF]

Additional file 1. Calculation of MS/MS FDRs.

We used the target/decoy approach to estimate the false discovery rates for the peptide-spectrum matches in this study. The decoy database was created by reversing the protein sequences in the target database. The target and decoy databases were searched separately by Mascot, OMSSA, and X! Tandem. However, it is necessary for InsPecT to filter the output in post-processing. The PValue.py script creates empirical *p*-values with a mixture model based on the percentage of decoy protein sequences in the concatenated database. The decoy database was appended to the target database to form the concatenated target/decoy database before the searches using InsPecT. While specifying a threshold of *E*-value/*p*-value of peptide identifications, the corresponding FDR can be estimated based on the number of peptide-spectrum matches from target (N_t) and decoy (N_d) database. The equation is N_d / N_t for the separate search strategy and 2 * N_d / (N_t + N_d) for the concatenated database strategy, respectively (Figure S1). By raising the threshold (lowering the *E*-value/*p*-value), the FDR was steadily reduced until < 2% (Supporting Table 1). For each algorithm, the search results which had a FDR < 2% were used in the comparison analysis.

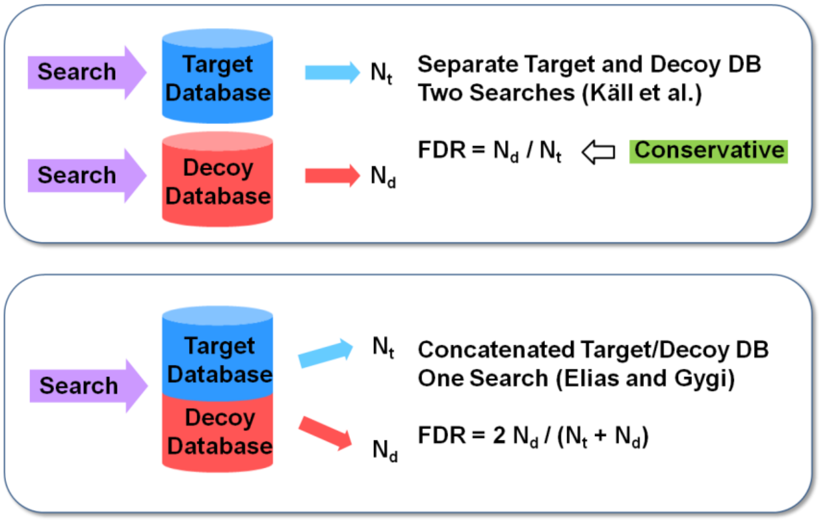

Figure S1. Two search approaches for FDR estimation using target and decoy databases.

Supporting Table 1: False discovery rates for peptide identifications with different search algorithms.

Algorithm	Threshold	Target/Decoy Database Search Approach	Number of Peptide-Spectrum Matches in		MS/MS FDR (%)
			Target DB	Reverse DB	
Mascot	*E* -value < 0.004	separated	11065	523	4.73
Mascot	*E* -value < 0.002	separated	10222	308	3.01
Mascot	*E* -value < 0.001	separated	9461	178	1.88
OMSSA	*E* -value < 0.10	separated	8461	174	2.06
OMSSA	*E* -value < 0.09	separated	8573	162	1.89
X! Tandem	*E* -value < 0.10	separated	10445	472	4.52
X! Tandem	*E* -value < 0.05	separated	9224	206	2.23
X! Tandem	*E* -value < 0.04	separated	8857	156	1.76
InsPecT	*p* -value < 0.05	concatenated	9706	223	4.49
InsPecT	*p* -value < 0.03	concatenated	9198	129	2.77
InsPecT	*p* -value < 0.02	concatenated	8832	85	1.91

References

1. Käll L, Storey JD, MacCoss MJ, Noble WS: **Assigning significance to peptides identified by tandem mass spectrometry using decoy databases.** *J Proteome Res* 2008, **7:**29-34.
2. Elias JE, Gygi SP: **Target-decoy search strategy for increased confidence in large-scale protein identifications by mass spectrometry.** *Nat Methods* 2007, **4:**207-214.
