# Supplementary material for: Identification of alternative splice variants in Aspergillus flavus through comparison of multiple tandem MS search algorithms
Source: BMC Genomics. 2011 Jul 11;12:358. doi: 10.1186/1471-2164-12-358 (PMC3146456; doi:10.1186/1471-2164-12-358)
Supplement: Additional file 2 — Comparison of overlapping identifications at different FDRs. Consensus decision of multiple search algorithms reached the similar overlaps regardless of the search results having the controlled or uncontrolled MS/MS FDRs. [file 1471-2164-12-358-S2.PDF]

Additional file 2. Comparison of overlapping identifications at different FDRs.

The calculation of the FDR is based on the *E*-value/*p*-value of the reported hits and a specified cut-off threshold. The statistic of the *E*-value or *p*-value is reported by the search engine. A stringent FDR may reduce the false positive hits, but discard the true identifications at the same time. To investigate whether the consensus decision approach is sensitive to the control of FDRs, we compared the identification overlaps between two sets of search results: FDRs of one set were uncontrolled and FDRs of the other set were kept under 2%. For each set of FDRs, the identified results were grouped by the number of algorithms which reported the same findings. The details of the comparison were summarized in Supporting Table 2. The illustration (Figure S2) of the comparison showed 1) the number of identifications decreased while requiring more tools having the same results; 2) the controlled-FDR results had less identifications than the uncontrolled-FDR results in general, but the difference diminished as the number of consensus increased; and 3) the identifications of peptides, RefSeq proteins and putative isoforms all followed a similar trend. This observation suggested that regardless of whether the consensus analysis started with the search results having the controlled or uncontrolled FDRs, the overlapping results of different search engines appeared more consistent as more algorithms concurred.

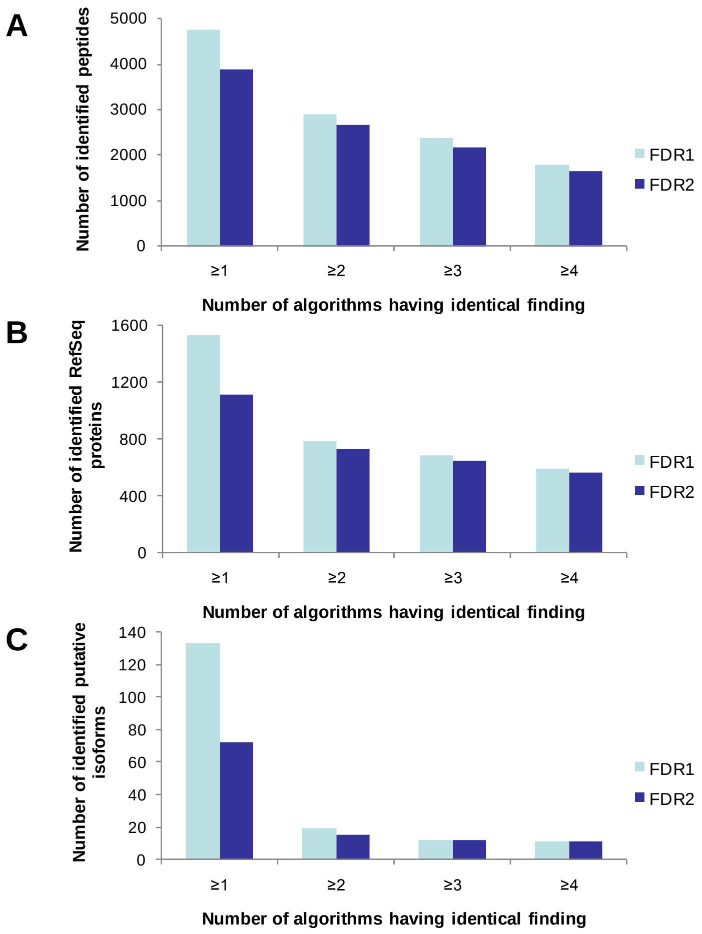

Figure S2. Consensus identifications while FDRs of search results are uncontrolled and controlled.

The number of the identified peptides (A), RefSeq proteins (B), and putative splice isoforms (C) were grouped by how many search algorithms reported the findings. The MS/MS FDRs of the search results were uncontrolled (FDR1) and controlled (FDR2) before the consensus analysis. The FDR1 group (light blue) representing the search results of Mascot, OMSSA, X! Tandem, and InsPecT, had a FDR of 4.73%, 2.06%, 4.52%, and 4.49%, respectively. In contrast, all search results in the FDR2 group (dark blue) had a FDR < 2%. FDR2 was equal to a FDR of 1.88%, 1.89%, 1.76, and 1.91% for the hits reported by Mascot, OMSSA, X! Tandem, and InsPecT, respectively.

Supporting Table 2: Number of peptides and proteins identified at different false discovery rates.

Algorithm	Peptides		RefSeq Proteins		Putative Isoforms	
	FDR1†	FDR2‡	FDR1†	FDR2‡	FDR1†	FDR2‡
Mascot only	538	275	209	58	30	6
OMSSA only	110	152	59	72	6	8
X! Tandem only	757	471	283	156	45	23
InsPecT only	461	334	193	98	33	20
Mascot, OMSSA	116	115	29	24	1	0
Mascot, X! Tandem	149	100	29	13	4	2
Mascot, InsPecT	102	80	18	10	1	1
OMSSA, X! Tandem	16	30	10	17	1	0
OMSSA, InsPecT	30	44	7	5	0	0
X! Tandem, InsPecT	118	108	10	12	0	0
Mascot, OMSSA, X! Tandem	203	185	44	36	0	0
Mascot, OMSSA, InsPecT	155	209	8	16	1	1
Mascot, X! Tandem, InsPecT	210	116	38	22	0	0
OMSSA, X! Tandem, InsPecT	9	21	6	7	0	0
Mascot, OMSSA, X! Tandem, InsPecT	1790	1651	589	568	11	11
Grand Total	4764	3891	1532	1114	133	72

† FDR1: 4.73% for Mascot; 2.06% for OMSSA; 4.52% for X! Tandem; and 4.49% for InsPecT.

‡ FDR2: 1.88% for Mascot; 1.89% for OMSSA; 1.76% for X! Tandem; and 1.91% for InsPecT.
